# Impact of schizophrenia genetic liability on the association between schizophrenia and physical illness: data-linkage study

**DOI:** 10.1192/bjo.2020.42

**Published:** 2020-11-10

**Authors:** Kimberley M. Kendall, Ann John, Sze Chim Lee, Elliott Rees, Antonio F. Pardiñas, Marcos Del Pozo Banos, Michael J. Owen, Michael C. O'Donovan, George Kirov, Keith Lloyd, Ian Jones, Sophie E. Legge, James T. R. Walters

**Affiliations:** MRC Centre for Neuropsychiatric Genetics and Genomics, Division of Psychological Medicine and Clinical Neurosciences, Cardiff University, UK; Health Data Research UK, Swansea University Medical School, Swansea University, UK; Health Data Research UK, Swansea University Medical School, Swansea University, UK; MRC Centre for Neuropsychiatric Genetics and Genomics, Division of Psychological Medicine and Clinical Neurosciences, Cardiff University, UK; MRC Centre for Neuropsychiatric Genetics and Genomics, Division of Psychological Medicine and Clinical Neurosciences, Cardiff University, UK; Health Data Research UK, Swansea University Medical School, Swansea University, UK; MRC Centre for Neuropsychiatric Genetics and Genomics, Division of Psychological Medicine and Clinical Neurosciences, Cardiff University, UK; MRC Centre for Neuropsychiatric Genetics and Genomics, Division of Psychological Medicine and Clinical Neurosciences, Cardiff University, UK; MRC Centre for Neuropsychiatric Genetics and Genomics, Division of Psychological Medicine and Clinical Neurosciences, Cardiff University, UK; Health Data Research UK, Swansea University Medical School, Swansea University, UK; National Centre for Mental Health, MRC Centre for Neuropsychiatric Genetics and Genomics, Division of Psychological Medicine and Clinical Neurosciences, Cardiff University, UK; MRC Centre for Neuropsychiatric Genetics and Genomics, Division of Psychological Medicine and Clinical Neurosciences, Cardiff University, UK; MRC Centre for Neuropsychiatric Genetics and Genomics, Division of Psychological Medicine and Clinical Neurosciences, Cardiff University, UK

**Keywords:** Schizophrenia, psychotic disorders, genetics, physical health

## Abstract

**Background:**

Individuals with schizophrenia are at higher risk of physical illnesses, which are a major contributor to their 20-year reduced life expectancy. It is currently unknown what causes the increased risk of physical illness in schizophrenia.

**Aims:**

To link genetic data from a clinically ascertained sample of individuals with schizophrenia to anonymised National Health Service (NHS) records. To assess (a) rates of physical illness in those with schizophrenia, and (b) whether physical illness in schizophrenia is associated with genetic liability.

**Method:**

We linked genetic data from a clinically ascertained sample of individuals with schizophrenia (Cardiff Cognition in Schizophrenia participants, *n* = 896) to anonymised NHS records held in the Secure Anonymised Information Linkage (SAIL) databank. Physical illnesses were defined from the General Practice Database and Patient Episode Database for Wales. Genetic liability for schizophrenia was indexed by (a) rare copy number variants (CNVs), and (b) polygenic risk scores.

**Results:**

Individuals with schizophrenia in SAIL had increased rates of epilepsy (standardised rate ratio (SRR) = 5.34), intellectual disability (SRR = 3.11), type 2 diabetes (SRR = 2.45), congenital disorders (SRR = 1.77), ischaemic heart disease (SRR = 1.57) and smoking (SRR = 1.44) in comparison with the general SAIL population. In those with schizophrenia, carrier status for schizophrenia-associated CNVs and neurodevelopmental disorder-associated CNVs was associated with height (*P* = 0.015–0.017), with carriers being 7.5–7.7 cm shorter than non-carriers. We did not find evidence that the increased rates of poor physical health outcomes in schizophrenia were associated with genetic liability for the disorder.

**Conclusions:**

This study demonstrates the value of and potential for linking genetic data from clinically ascertained research studies to anonymised health records. The increased risk for physical illness in schizophrenia is not caused by genetic liability for the disorder.

## Background

Individuals with schizophrenia have a 20-year reduced life expectancy.^1^ A major contributing factor is the increased rate of poor physical health outcomes in individuals with schizophrenia, related to conditions such as metabolic, cardiovascular and respiratory disease.^2^ Identifying the underlying reasons for these health disparities will provide a first step towards closing this health gap and thus has become a priority in schizophrenia research and clinical care in recent years.^3^ It is unclear whether the poorer physical health outcomes in schizophrenia (a) arise from the pleiotropic action of schizophrenia risk factors or (b) are secondary to illness-related factors such as negative symptoms, adverse effects of treatment (particularly antipsychotic medication) or poorer access to healthcare.

Genetic factors make major contributions to schizophrenia risk, arising from both common genetic variation and rare variants such as copy number variants (CNVs), the latter of these also being associated with physical health consequences in population samples.^4,5^ A recent study, in which genetic and electronic health record data were linked for 106 160 individuals, reported associations between polygenic risk scores for schizophrenia and several physical health phenotypes including smoking and reduced rates of obesity.^6^ At present, it is not known whether these genetic risk factors for schizophrenia are also associated with physical ill health in those with the disorder.

One reason for the dearth of research examining genetics and physical health outcomes in those with mental health disorders is likely to be the lack of available physical health data in existing psychiatric cohorts. In contrast, linkage of national registry data to anonymised mental and physical healthcare records, as exemplified in Nordic countries, has provided a rich resource for mental health research and led to important insights including into physical health outcomes in schizophrenia.^7,8^ The use of anonymised National Health Service (NHS) records and linkage of patient data to national registries in the UK is gaining momentum^9,10^ and this approach has enabled researchers to investigate healthcare-related outcomes for individuals with major mental health disorders such as schizophrenia.^2^ The amalgamation and linkage of health record and genomic data-sets offers further potential and initial reports describing linkage are emerging.^6,11–13^ This type of approach has the potential to facilitate the collection of large-scale phenotypic data without the need to burden patients with extensive assessments. This may have particular advantage for physical health outcomes, which could be tracked longitudinally with updated health record linkage.

## Study aims

In this study, we aimed to link genetic data from a clinically ascertained sample of individuals with schizophrenia to anonymised NHS and administrative data-sets in the Secure Anonymised Information Linkage (SAIL) databank in Wales,^9^ with a focus on the rich physical health outcome data held in primary care electronic health resources. We then aimed to examine the association between physical health outcomes and genetic liability for schizophrenia as indexed by (a) rare (frequency <1%) CNVs, and (b) polygenic risk scores.

## Method

### Participants

Study individuals (*n* = 958, aged 17–84 years, 41% female) from the Cardiff Cognition in Schizophrenia (CardiffCOGS) sample were recruited from community, in-patient and voluntary sector mental health services in the UK and underwent detailed phenotypic assessment including a Schedules for Clinical Assessment in Neuropsychiatry (SCAN) interview.^14^ Interview data and clinical case-note vignettes were then used to arrive at a best estimate lifetime diagnosis according to DSM-IV criteria.^15^ Ethical approval was obtained from relevant NHS multisite research ethics committees and written informed consent was obtained for all study participants for genetic research and linkage of their genetic information to the SAIL databank. Further information on the CardiffCOGS sample has been published elsewhere.^16^

### Electronic cohort from SAIL

#### Data source and data linkage

The SAIL databank (http://www.saildatabank.com) is a national data repository containing anonymised, person-based, linkable data in Wales for over 3 million people. The procedure for linking research study data to SAIL has been described elsewhere.^9,17,18^ In brief, data from CardiffCOGS study individuals who had consented to linkage were imported into SAIL in line with permissions already granted to SAIL relating to good practice in research governance and privacy protection. We adopted a split file approach to separate individual identifiers from the interview data. Identity matching and creation of pseudonymised linkage keys (anonymised linking fields) were performed by a trusted third party prior to linkage and further encryption of data sets using deterministic matching based on NHS number or probabilistic matching using available demographics based on the Welsh Demographic Service data-set (all individuals registered with a general practice (GP) surgery). We included participants whose data were probabilistically linked with an adequate level of matching accuracy (matching score ≥0.9).^18^

We used the General Practice Database (GPD), containing diagnoses, symptoms, investigations, prescribed medication, referrals, coded hospital contacts and test results. At time of analysis, 77% (333/432) of GP surgeries in Wales supplied their data to SAIL. We also extracted data from the Patient Episode Database for Wales (PEDW), an NHS Wales hospital admissions data-set consisting of clinical information from all hospital admissions (in-patient and day cases) covering the entire population of Wales.

#### Measures for health outcomes

We used ICD-10 and Read codes for GDP and PEDW data-sets, respectively, to ascertain health outcomes.^19^ For schizophrenia, we adopted the codes that were validated and used in previous studies.^18,20,21^ We selected smoking, type 2 diabetes mellitus, ischaemic heart disease and body mass index (BMI) because of their high frequency in clinical samples of individuals with schizophrenia and their potential to contribute to increased mortality either directly, or via phenomena such as metabolic syndrome. We selected congenital disorders, intellectual disability and epilepsy as they are neurodevelopmental phenotypes with direct relevance to CNV carrier status. All of these variables also had either established SAIL algorithms or were considered to have high-quality data available in SAIL. We identified individuals with intellectual disability,^22,23^ ischaemic heart disease,^17^ epilepsy,^24^ diabetes mellitus^25^ and determined smoking status^26^ based on previously published works.

The list of codes used for extracting height, BMI and for identifying individuals with congenital disorders are given in supplementary Table 1 available at https://doi.org/10.1192/bjo.2020.42. For identifying schizophrenia, intellectual disability, ischaemic heart disease, epilepsy, diabetes mellitus and congenital disorders, we combined both the GPD and PEDW data-sets. We additionally extracted individuals’ height, BMI and smoking status from the GPD where available.

For comparison, we extracted lifetime diagnoses and estimated crude rates, as well as age- and gender-standardised rates, of health outcomes for the whole population and those diagnosed with schizophrenia between the ages of 17 and 84 years (as at 30 June 2016). For estimating standardised rates from SAIL, crude rates were first computed for five age groups (17–34, 35–44, 45–54, 55–64 and 65–84 years) for both genders. Standardised rates were then estimated based on the age and gender distribution in the clinical cohort. Linked data in SAIL were interrogated using structured query language (SQL DB2).

### Genetic data

#### Genotyping and CNV calling

Samples were genotyped on OmniCombo or OmniExpress arrays (Illumina).^16^ After standard quality control, imputation was performed using IMPUTE2^27^ and the 1000 genomes (phase 3) and UK10 K reference panels.^28^ Best-guess genotypes were generated with the following thresholds: minimal genotypic confidence >90%, INFO-score >0.9, minor allele frequency (MAF) >1%, and Hardy-Weinberg equilibrium *P-*value <1 × 10^−10^.

Full details of the CNV calling methods and quality controls measures used have been published elsewhere.^29^ Illumina Genome Studio (version 2011.1) was used to process raw intensity data into log R ratios (LRR) and B allele frequencies. PennCNV (version 1.0.3.18) was then used to call CNVs based on 666 868 probes common to all Illumina arrays used.^30^ CNVs were joined if separated by less than 50% of their combined length. CNVs were excluded if they were (a) called with fewer than ten probes, (b) overlapped low copy repeats by more than 50% of their length, (c) had a probe density of less than 1 probe/20 kb, or (d) had a frequency of >1%.^31^

#### CNVs

To examine for enrichment of rare, pathogenic CNVs in physical health comorbidity, we analysed the presence of 12 CNVs robustly associated with the risk of schizophrenia,^16,29^ and 54 CNVs nominally associated (*P* < 0.05) with intellectual disability, autism spectrum disorder or schizophrenia (‘neurodevelopmental CNVs’).^32^ Following the approach taken in our previous work, 15q11.2 duplications were excluded because of their high frequency.^33^ CNV burden analyses were carried out using PLINK on regions of variable copy number at two size thresholds (a) ≥500 KB and (b) ≥1 MB and converted into carrier status for the purpose of regression analyses.

#### Polygenic risk scores

Polygenic risk scores were calculated using the largest published schizophrenia genome-wide association study meta-analysis (39 915 individuals with schizophrenia, 64 639 controls) as a training set and using the established method described by Wray et al.^34,35^ All study individuals were excluded from the training set. scores were generated using the –score function in PLINK^31^ for Single nucleotide polymorphisms with MAF >10%, INFO score >0.9, a low linkage disequilibrium to each other and excluding indels and the extended major histocompatibility complex region. Polygenic risk scores were calculated at nine *P*-value thresholds; 1 × 10^−8^, 1 × 10^−6^, 1 × 10^−4^, 1 × 10^−3^, 0.01, 0.05, 0.1, 0.2 and 0.5.

### Analysis

#### Rates of physical illness

Linked data in SAIL were interrogated using structured query language (SQL DB2). All crude rates and standardised rates of health outcomes were expressed as a percentage of population affected (lifetime prevalence). All standardised rate ratios (SRRs) and their 95% confidence intervals were calculated as previously described.^36–38^

#### Ascertainment rates of behaviours and diagnoses

We evaluated the agreement on diagnoses between the clinical and electronic cohorts for each health outcome by constructing two × two contingency tables based on the paired responses from the interview and SAIL. Level of agreement was then assessed by unweighted Cohen's kappa coefficient^39^ and Gwet's AC1.^40^ The 95% confidence intervals of Cohen's kappa and Gwet's AC1 were estimated as described in Fleiss, Cohen & Everitt^41^ and Gwet,^40^ respectively. Strength of agreement metrics was categorised according to previously described criteria.^42^

#### CNV analyses

Association analyses were carried out using linear regression for average BMI (normalised using log_10_ transformation) and average height, and Firth logistic regression for all binary traits (type 2 diabetes mellitus, smoking, ischaemic heart disease, congenital disorders, epilepsy, intellectual disability). Firth logistic regression allows for the analysis of low numbers in addition to the inclusion of covariates. All analyses included age and gender as covariates.^43^

#### Polygenic risk analyses

We regressed a model for each polygenic risk score created from various training *P*-value thresholds against a base model including age, gender, the first five principal components and any additional principal components from the first 20 that were nominally associated with the phenotype of interest. We repeated these analyses with the addition of covariates reflecting symptom severity, non-response to antipsychotics, antipsychotic exposure, smoking status and genotyping platform (defined in supplementary Table 2). All statistical analyses were carried out in R and results were subject to Bonferroni correction for the eight phenotypes examined (*P-*value threshold 0.0063).

## Results

A total of 896 (93.5%) study individuals from CardiffCOGS were linked to health records held in the SAIL databank. Linked study individuals had an age range of 17–84 years (mean 44 years), 371 (41%) were female and 724 (81%) had genetic data available.

### Physical health outcomes in CardiffCOGS and SAIL

**Fig. 1:**
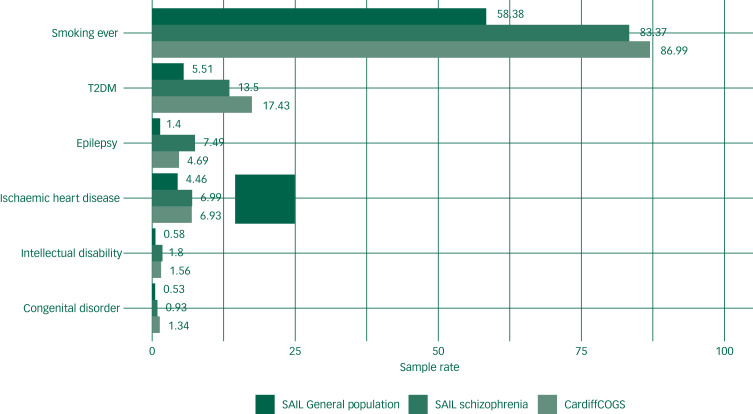
Rates of physical health phenotypes in the Cardiff Cognition in Schizophrenia (CardiffCOG) schizophrenia sample, the schizophrenia population in Secure Anonymised Information Linkage (SAIL) and the general population in SAIL. T2DM, type 2 diabetes mellitus.

Table 1 and Fig. 1 outlines the frequencies of the physical health outcomes in the CardiffCOGS sample and SAIL. Within the SAIL population, individuals with schizophrenia had increased rates of epilepsy (SRR = 5.34, 95% CI 5.11–5.57), intellectual disability (SRR = 3.11, 95% CI 3.06–3.11), type 2 diabetes (SRR = 2.45, 95% CI 2.38–2.53), congenital disorders (SRR = 1.77, 95% CI 1.57–1.99), ischaemic heart disease (SRR = 1.57, 95% CI 1.51– 1.63) and smoking (SRR = 1.44, 95% CI 1.42–1.46). Individuals within CardiffCOGS had higher rates of type 2 diabetes (SRR = 1.29, 95% CI 1.10–1.52) compared with the population with schizophrenia in SAIL. Crude unadjusted population rates are given in supplementary Table 3.

**Table 1:** Rates of physical health phenotypes in Cardiff Cognition in Schizophrenia (CardiffCOG) sample compared with the population rates in Secure Anonymised Information Linkage (SAIL)^a^

Phenotype	CardiffCOGS rate, % (*n*)	SAIL schizophrenia population rate, % (*n*)	SAIL population rate, % (*n*)	SRR_sam, sch_ (95% CI)	SRR_sch, gen_ (95% CI)
Congenital disorder	1.34 (12)	0.93 (326)	0.53 (21 745)	1.44 (0.81–2.57)	1.77 (1.57–1.99)
Intellectual disability	1.56 (14)	1.80 (623)	0.58 (22 142)	0.87 (0.51–1.48)	3.11 (3.06–3.11)
Ischaemic heart disease	6.93 (62)	6.99 (3449)	4.46 (207 197)	0.99 (0.77–1.28)	1.57 (1.51–1.63)
Epilepsy	4.69 (42)	7.49 (2628)	1.40 (52 020)	0.63 (0.46–0.85)	5.34 (5.11–5.57)
Type 2 diabetes mellitus	17.43 (156)	13.50 (5293)	5.51 (216 187)	1.29 (1.10–1.52)	2.45 (2.38–2.53)
Smoking (current/ex)	86.99 (689)	83.87 (22 120)	58.38 (1 649 589)	1.04 (0.97–1.11)	1.44 (1.42–1.46)

sam – sample (CardiffCOGS), sch – schizophrenia, gen – general population.

a. SRR_sam, sch_ represents the standardised rate ratio (SRR) of the clinical cohort to the schizophrenia population ascertained in SAIL. SRR_sch, gen_ represents the standardised rate ratio of the schizophrenia population ascertained in SAIL to the general population ascertained in SAIL. SAIL rates were standardised using the age and gender distribution from the clinical cohort as reference. Crude unadjusted population rates are given in supplementary Table 3. Numbers stated are out of 895 for the CardiffCOGS sample (except for smoking which was out of 792). Numbers stated for the population rate are out of 3 852 471 (except for smoking which was out of 2 958 064) and for schizophrenia population are out of 35 944 (26 588 for smoking). Rates given in parentheses in SAIL columns are standardised to account for differences in age and sex distribution between the SAIL and CardiffCOGS cohorts.

### Comparison of ascertainment rates of behaviours and diagnoses in CardiffCOGS and SAIL

For physical health outcomes that were also reported at interview (smoking, type 2 diabetes mellitus, ischaemic heart disease, and epilepsy), we compared agreement with their health records (Table 2). Both Cohen's κ and Gwet's AC1 for all physical conditions ranged from 0.502 to 0.936. These show that the agreement of the rates ascertained from the interview and health records were moderate to high.^42^ The highest agreements were observed for ischaemic heart disease and epilepsy. The strength of agreement was lowest for smoking behaviour (Cohen's κ = 0.380 and Gwet's AC1 = 0.621), reflecting only fair to substantial agreement.

**Table 2:** Interrater agreement of the rates of ascertainment between the Cardiff Cognition in Schizophrenia (CardiffCOG) interview and linkage to National Health Service records^a^

	CardiffCOGS interview		% Reported affected at CardiffCOGS interview	% Reported affected in SAIL		
SAIL	Yes	No			Cohen's κ (95% CI)	Gwet's AC1 (95% CI)
T2DM						
Affected	75	43	13.34	17.69	0.675 (0.597–0.754)	0.884 (0.853–0.915)
Unaffected	14	535				
Ischaemic heart disease						
Affected	21	19	5.96	6.12	0.502 (0.362–0.641)	0.936 (0.915–0.957)
Unaffected	18	596				
Epilepsy						
Affected	24	10	7.81	5.11	0.529 (0.397–0.661)	0.935 (0.914–0.956)
Unaffected	28	604				
Smoking						
Affected	515	119	71.92	77.41	0.380 (0.307–0.452)	0.621 (0.567–0.675)
Unaffected	74	111				

SAIL, Secure Anonymised Information Linkage.

a. The smoking variable compares results for current/ex smoking status in SAIL and ever a regular smoker at interview. Cohen's κ refers to Cohen's kappa statistic,^45^ Gwet's AC1 refers to the first order agreement coefficient.^46^ Both values range between 0 (chance, or no agreement) and 1 (perfect agreement). Values below 0.4 indicate poor agreement, values of 0.4–0.59 indicate moderate agreement and values above 0.6 indicate substantial agreement.

### CNV

A total of 2.1% (*n* = 15) of the CardiffCOGS sample carried a schizophrenia-associated CNV, 4.9% (*n* = 32) carried a neurodevelopmental CNV, 9.5% carried a chromosomal duplication or deletion ≥500 KB (19 deletions, 52 duplications, 2 both) and 3.2% carried a duplication or deletion ≥1 MB (6 deletions, 17 duplications). We found no evidence that CNV carriers had increased rates of poor physical health outcomes (Table 3). However, average height was nominally associated with carrier status for CNVs associated with schizophrenia (beta = −0.075, 95% CI −0.14 to −0.01, *P* = 0.017) and neurodevelopmental disorders (beta = −0.077, 95% CI −0.64 to −0.07, *P* = 0.015) (Table 3). CNV carriers were on average 7.5–7.7 cm shorter than non-carriers. There was no evidence for association between rare CNVs of 500 KB or greater and any of the phenotypes examined (supplementary Table 4).

**Table 3:** Association analysis results for physical health phenotypes and two groups of copy number variants (CNVs, 12 schizophrenia CNVs and 53 neurodevelopmental CNVs)^a^

Phenotype	Schizophrenia CNVs				Neurodevelopmental CNVs			
	Carriers	Non-carriers	Effect size (95% CI)	*P*	Carriers	Non-carriers	Effect size (95% CI)	*P*
Average height (GP)	13	611	–0.075 (–0.14 to –0.01)	0.017	31	593	–0.077 (–0.64 to –0.07)	0.015
Average BMI log_10_ (GP)	13	604	0.04 (–0.03 to 0.09)	0.32	30	587	0.02 (–0.26 to 0.48)	0.56
Type 2 diabetes mellitus (both)								
Affected	<5	126	0.45 (–0.79 to 1.53)	0.45	6	124	–0.15 (–1.12 to 0.68)	0.74
Unaffected	11	576			30	557		
Smoking – current or ex-smoker (GP)								
Affected	9	552	–0.85 (–1.86 to 0.21)	0.11	25	536	–0.46 (–1.15 to 0.29)	0.23
Unaffected	<5	78			7	75		
Ischaemic heart disease (both)								
Affected	0	53	–1.15 (–6.03 to 0.99)	0.36	<5	52	–1.08 (–3.31 to 0.35)	0.16
Unaffected	15	656			35	636		
Congenital disorders (both)								
Affected	<5	8	1.97 (–0.32 to 3.59)	0.08	<5	8	1.29 (–0.97 to 2.83)	0.21
Unaffected	14	701			35	680		
Epilepsy (both)								
Affected	<5	36	1.23 (–0.43 to 2.48)	0.13	<5	34	0.94 (–0.24 to 1.89)	0.11
Unaffected	13	673			32	654		
Intellectual disability (both)								
Affected	<5	8	2.13 (–0.17 to 3.73)	0.06	<5	8	1.26 (–0.99 to 2.79)	0.22
Unaffected	14	701			5	680		

*P*, uncorrected *P*-value; GP, general practice.

a. General practice/hospital/both in parentheses in the phenotype column refers to the source of diagnostic information used. Effect size refers to odds ratio in all cases except average height and average body mass index for which the effect size is standardised Beta. Counts below five are masked to preserve participant anonymity.

### Polygenic risk for schizophrenia

**Fig. 2:**
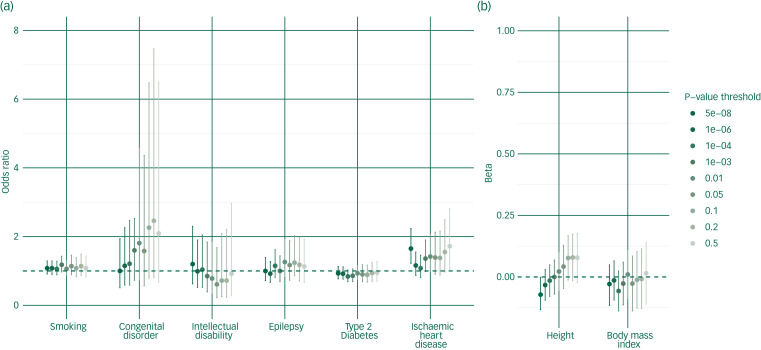
Graphs of the results from regression models for the association between polygenic risk for schizophrenia and physical health outcomes. (a) Odds ratio; (b) Beta. Odds ratios are shown for the *P*-value thresholds at which markers were selected.

We found no evidence for an association between polygenic risk scores for schizophrenia and the physical health outcomes studied (Fig. 2, supplementary Table 5), although there was weak evidence for an association with ischaemic heart disease at the genome-wide *P*-value threshold (odds ratio (OR) = 1.65, 95% CI 1.22–2.24; adjusted *R*^2^ = 0.035, *P* = 0.001). The lack of association between schizophrenia polygenic risk scores and physical health outcomes remained in sensitivity analyses covarying for symptom severity, non-response to antipsychotics, antipsychotic exposure, smoking status and genotyping array (supplementary Table 6). However, we did identify significant associations between non-response to antipsychotics and type 2 diabetes (OR = 2.94, 95% CI 1.79–4.85, *P* ≤ 0.0001) and an association between symptom severity and intellectual disability (OR = 1.24, 95% CI 1.05–1.46, *P* = 0.0012). There were additional nominal associations (*P* < 0.05) between non-response to antipsychotics and epilepsy, and smoking and ischaemic heart disease (supplementary Table 6).

## Discussion

In this study, we report the linkage of genetic data from a clinically ascertained sample of individuals with schizophrenia to anonymised NHS health records in the SAIL databank. Consistent with data collected at interview, we found that individuals with schizophrenia in Wales had increased rates of neurodevelopmental disorders (epilepsy, intellectual disability and congenital disorders), smoking, type 2 diabetes mellitus and ischaemic heart disease compared with the general population. However, the results of our genetic analyses suggest that these increased rates are not because of genetic liability to schizophrenia; we found no evidence for an association between genetic risk for schizophrenia indexed by CNVs or polygenic risk scores and physical health outcomes.

These findings are supported by a recent population-based study that linked genetic and health record data for 106 160 individuals and found an inverse association between increased schizophrenia polygenic risk scores and obesity and an inverse association with diabetes when controlling for schizophrenia diagnosis or for antipsychotic medication history.^6^ No association was found between schizophrenia polygenic risk scores and the other physical health outcomes found in this study.^6^ Thus, the evidence available indicates that increased rates of poor physical health observed in patients with schizophrenia is unlikely to be driven by the genetic liability for the disorder.

There may be many other factors contributing to an increased rate of the physical health outcomes examined. For the non-neurodevelopmental outcomes, these include, but are not limited to, medication side-effects and lifestyle choices such as smoking and diet. For example, weight gain caused by antipsychotic medication, poor diet and smoking may all contribute to the risk of type 2 diabetes mellitus and ischaemic heart disease. This is supported by our study; we found significant associations between non-response to antipsychotics and type 2 diabetes mellitus and nominal associations between non-response to antipsychotics and epilepsy, and smoking and ischaemic heart disease. The associations with non-response to antipsychotics may reflect the frequent use of clozapine in this patient group, an antipsychotic known to cause weight gain and increase the risk of type 2 diabetes mellitus. Our additional analyses also found that intellectual disability was significantly associated with a greater symptom severity. The reasons for this association are not yet clear. Importantly, several of these non-genetic factors are amenable to targeted intervention, which may reduce the risk of their consequent physical health outcomes.

Our finding that carriers of rare neurodevelopmental CNVs tend to be shorter than CNV non-carriers is in keeping with previous work on CNV associations with anthropometric traits in a sample of 191 161 adults.^44^ Macé et al report associations between both total CNV burden, several individual CNV loci (also examined in our study) and alterations in height. Additional findings from Macé et al of a lack of association between anthropomorphic traits and schizophrenia suggest that the CNVs affect height independent of disease.^44^

The rates of smoking ascertained in our study (87.0%) and in those with schizophrenia in SAIL (83.9%) appear high but are comparable with several studies also examining ‘ever’ smoking in individuals with schizophrenia.^45^ Similarly, the rates of ischaemic heart disease in the CardiffCOGS and SAIL schizophrenia population groups (both 6.9%) are comparable with rates established in other studies.43,44 CardiffCOGS had a higher rate of type 2 diabetes mellitus (17.4%) than the schizophrenia population in SAIL (13.5%) and other study samples such as a Swedish cohort study of individuals with schizophrenia (11–12.5%).^1^ This may reflect the higher than average proportion of individuals with chronic schizophrenia and long-term antipsychotic medication use in the CardiffCOGS sample.

### Study limitations

The main limitation of this study was related to sample size; it is possible that we were underpowered to detect genetic associations with small effect sizes. However, it can still be concluded that genetic liability to schizophrenia does not have a large or significant impact on the occurrence of physical comorbidity. Nonetheless, our plan for future work is to link genetic data for a far greater number of individuals to their health records. This study does provide an important exemplar for the value of linking genetic data to routinely collected health-related data. Such an approach has great potential to generate a wealth of evidence, which can be translated into improved health outcomes for patients.

## Data Availability

All data requests should be submitted to the corresponding authors for consideration.

## Supplementary material

For supplementary material accompanying this paper visit http://dx.doi.org/10.1192/bjo.2020.42. click here to view supplementary material
